# Potential of Neglected and Underutilized Yams (*Dioscorea* spp.) for Improving Nutritional Security and Health Benefits

**DOI:** 10.3389/fphar.2020.00496

**Published:** 2020-04-24

**Authors:** Bandana Padhan, Debabrata Panda

**Affiliations:** Department of Biodiversity and Conservation of Natural Resources, Central University of Orissa, Koraput, India

**Keywords:** ethnobotany, nutritional security, pharmacological properties, tuber yield, wild yam

## Abstract

Food and nutritional security are the major concerns in many countries of the world and may have potential to contribute to sustainable food systems under climate change. To address the food and nutritional insecurity, it has become extremely important to diversify the present-day agricultural system as well as to search for alternative food and feed ingredients. Some wild root and tuber crops occupy a remarkable position toward food security of the developing world due to their high calorific value and superior carbohydrate content. Yam (*Dioscorea* spp.) provides food and medicines to millions of people in the world especially in the tropics and sub tropics. It is recognized as the fourth most important tuber crop after potatoes, cassava, and sweet potatoes. It contributes about 10% of the total root and tubers production around the world. Yams also considered as famine food and plays a prime role in the food habit of small and marginal rural families and forest-dwelling communities during the food scarcity periods. These species are unique for their food, medicinal and economic values but their wider utilization is limited due to the presence of anti-nutritional compositions. This paper describes the ethnobotany of yam species in relation to their nutritional, anti-nutritional and pharmacological properties and highlights the potentiality for food and nutritional security for combating the “hidden hunger” caused by micronutrient deficiencies.

## Introduction

Yam (*Dioscorea* spp.) is considered as a famine food and plays a prime role in the food habit of small and marginal rural families and forest-dwelling communities during the food scarcity periods (Ngo Ngwe et al., 2015). It is recognized as the fourth most important tuber crop after potatoes, cassava, and sweet potatoes and contributes about 10% of the total root and tubers production around the world (Viruel et al., 2016). *Dioscorea* tubers have nutritional advantage over other root crops (Shajeela et al., 2011). It contains good source of essential dietary supplements such as protein, well balanced essential amino acids, and many dietary minerals (Baah et al., 2009). *Dioscorea* species are the monocotyledonous tuber crops under family Dioscoraceae and the genus includes more than 600 different species worldwide (Amanze et al., 2011). Most of the species are unique for their food, medicinal and economic values but their wider utilization is limited due to the presence of anti-nutritional compositions. There is an enormous diversity in the wild and domesticated species that are being used by tribal communities as traditional food. However, systematic characterization of food quality traits in wild species is a major prerequisite for mass consumption and cultivation. The characterization of wild yam species both at phenotypic and molecular level is a major prerequisite for identification of better yam genotypes with improved traits to integrate them in future yam breeding program (Arnau et al., 2017). An acquisition of understanding of the chemical properties of the wild tubers may help for bio-prospecting of the tuber in food industries. Despite its economic and cultural importance, breeding, and selection of yam genotypes with improved traits is currently inhibited by the lack of adequately characterized wild species both at the morphological and molecular level. The dearth of knowledge regarding population structure has significantly contributed to genetic erosion of yams. Therefore, the present review describes the ethnobotany of wild yam species in relation to their nutritional, anti-nutritional, and also highlights the recent progress in pharmacological properties of wild yam species for addressing future food and nutritional security.

## Origin and Distribution of *Dioscorea*

The genus *Dioscorea* is considered as one among the earliest angiosperms originated from Southeast Asia and Indo-Malayan region (Kumar et al., 2017). The major yam species are available in three isolated regions of the World: Southeast Asia, Tropical America, and West Africa (Kumar et al., 2017). These regions are major yam growing centers of the world and represent considerable diversity (Kumar et al., 2017). Out of 600 yam species, only seven are mostly consumed in West Africa, 93 species, and nine varieties are found in China and 14 species and five varieties found in Taiwan (Price et al., 2017). Of these, seven to ten are cultivated species and two (*D. alata* L., *D. cayennensis* Lam. subsp. *cayennensis* and *D. cayennensis* Lam. subsp. *rotundata* (Poir.) J. Miège) are of primary importance as a staple crop, predominately in Western Africa, for over 100 million people (Price et al., 2018). Approximately 50 species are consumed as wild-harvested staples or famine food. The most well-known species is *Dioscorea villosa* L., also called wild yam and is native to North America (Avula et al., 2014). The cultivated species *Dioscorea esculenta* (Lour.) Burk was known to originated from China. *D. alata* L. is the most economically important species originated in Southeast Asia specifically, in Tropical Myanmar and Thailand (Tamiru, 2006), and is most diversified and extensively distributed species throughout tropical Asia and the Pacific. *D. bulbifera* L. is the most popular wild *Dioscorea* species is native to Asia, tropical Africa, and Northern Australia (Kumar et al., 2017). Another wild species, i.e., *D. pubera* Blume is native to the Indo-China region (Kumar et al., 2017) and distributed throughout the temperate, tropical Americas, China, wet regions of Himalayas, Central Nepal, Western Malaysia, and Bhutan (Kumar et al., 2017). Whereas *D. pentaphylla* L. is native to Tropical Asia and Eastern Polynesia and is distributed in South-Eastern Asia, Tropical Asia, North America (Kumar et al., 2017). In India, there are more than 50 different species of *Dioscorea* reported in the states like Assam, Tamilnadu, Kerala, Bihar, Odisha, West Bengal, Rajasthan, Gujaratm and Maharashtra (Kumar et al., 2017). Out of 50 species recorded, maximum number of yam species have been recorded from Assam (19) followed by Tamilnadu (16) and Darjeeling and Sikkim (15) (Kumar et al., 2017). The wild species *D. prazeri* Prain & Burkill and *D. deltoidea* Wall. ex Griseb.are found at high altitudes (Saikia et al., 2010). The *Dioscorea* species such as *D. belophylla* (Prain) Voigt ex Haines, *D. wightii* Hook.f. and *D. spicata* Roth are endemic to Western Ghats (Kumar et al., 2017).

## Botany of *Dioscorea* Species

**Figure 1:**
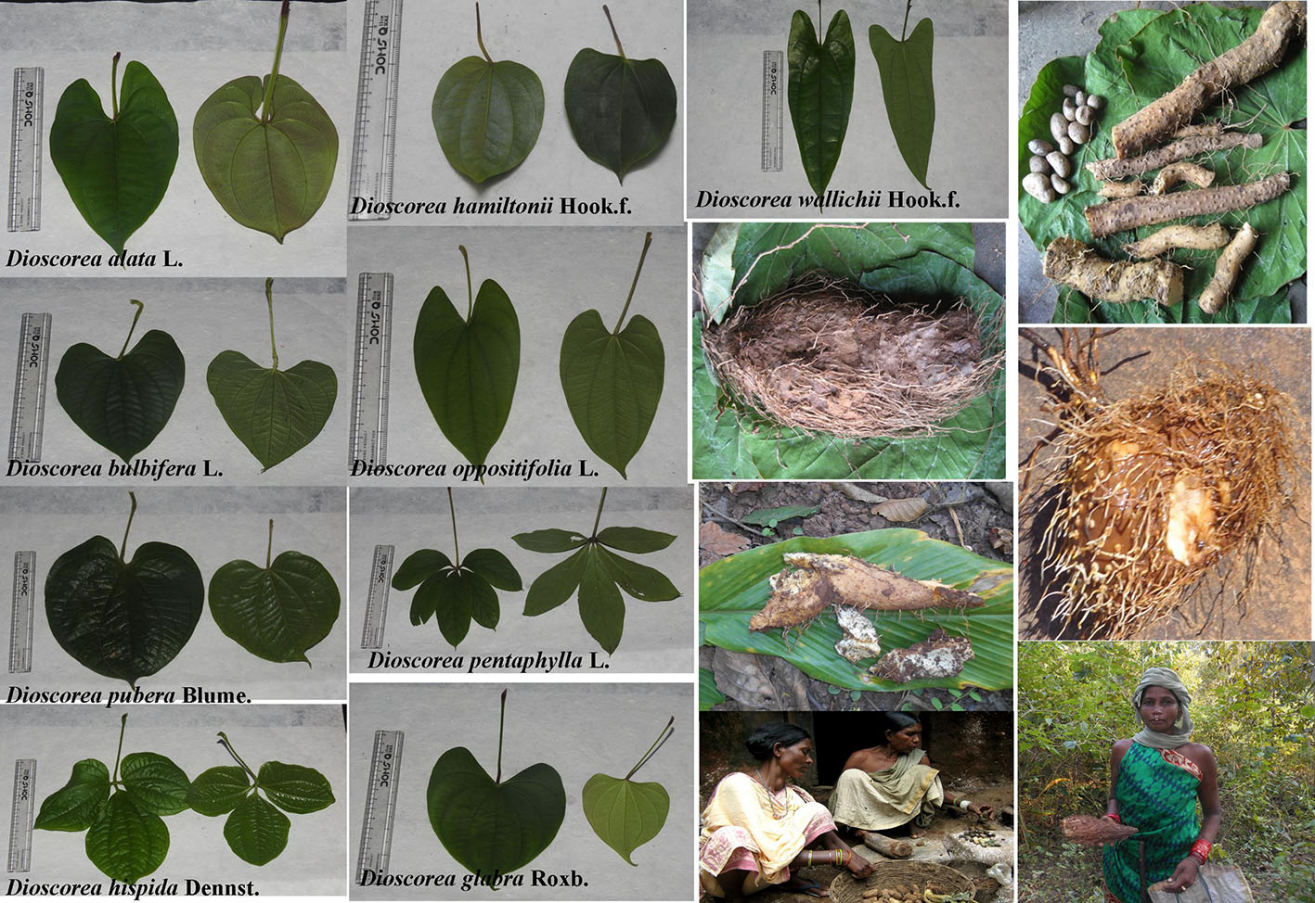
Morphological variations of different *Dioscorea* species consumed by the tribal people.

Yam, a monocotyledons plant of *Dioscorea* genus under Dioscoreaceae family belongs to the order Dioscoreales under the division Magnoliophyta (Kumar et al., 2017). The wild species of *Dioscorea* are either annual or semi-perennial or perennial whereas most of the cultivated species are annuals. The leaves of some species are cordate, simple or acuminate having long petiole and some species have palmate or lobed having pointed tips. All the *Dioscorea* species are climber and climbs by twining the stem. The direction of stem twining (i.e., left to right or right to left) of *Dioscorea* species is a peculiar characteristic for identification of species within the genus. Some right twining wild *Dioscorea* species are *D. oppositifolia* L.*, D. hamiltonii* Hook.f.*, D. pubera, D. wallichii* Hook.f., and *D. glabra* Roxb., which have simple leaves and the compound leave *Dioscorea* species are *D. hispida* Dennst. and *D. pentaphylla* are left twiner species (Behera et al., 2009) (**Figure 1**). Most of the commercially cultivated *Dioscorea* species such as *D. alata*, *D. rotundata*, *D. opposite* L., *D. cayennensis* and *D. japonica* Thunb. are placed under Enantiophyllum section (Peter, 2007). Other cultivated species such as *D. esculenta* is placed under Combilium section, *D. trifida* L.f. in Macrogynodium, *D. dumetorum* (Kunth) Pax belongs to Lasiophyton (Peter, 2007). The stem wings are present in some species especially in cultivated species (*D. alata*), which helps in twinning of the vine. The flowers of the *Dioscorea* species are dioecious in nature, having male and female flowers present separately or on separate plants. The male or female flowers grow on the axillary spikes of the leaf axils. The male flowers are glabrous, sessile and spherical which are borne axially or terminally. The fruits are mostly small capsules with wings and the shape varies in different species (Behera et al., 2009). The seeds are light and flat in shape, the wings help for seed dispersion. Some *Dioscorea* species *i.e. D. bulbifera*, *D. alata*, *D. pentaphylla*, and *D. pubera* have bulbils grown in the axils. These bulbils are used as planting materials. Tubers of *Dioscorea* species are shallow, fibrous and mostly unbranched. Most of the tubers are placed in the top layer of the soil and some are deeply buried up to 1 m depth (Behera et al., 2009; Kumar et al., 2017). The tubers are the storage organ for carbohydrates. The new tuber formation and shrivels of the old one occurs simultaneously when the re-growth is initiated.

## Ethno-Botanical Studies of *Dioscorea*

The term “Ethno-botany” is a multidisciplinary science that deals with the study of how the people of an exacting culture and region make use of plants. Different *Dioscorea* species plays a remarkable position in the traditional medicines for the treatment of various diseases (Kumar et al., 2017). There are numerous reports available on ethno-medicinal uses of different *Dioscorea* species worldwide (Mishra et al., 2008; Sharma and Bastakoti, 2009; Sheikh et al., 2013). In South Asia, the tuber syrup is used to reduce labour pain and to treat various diseases such as colic pain, asthma, cough, rheumatism, and gastric problem (Foster and Duke, 2000). The tuber extract of *D. deltoidea* Wall. is used to treat urino-genital disorders, helminthes infection, and constipation (Gangwar and Joshi, 2008). The native people of Southern Thailand use yam tubers to cure warts (Maneenoon et al., 2008). The tuber mucilage of *Dioscorea borneensis* R.Knuth (syn. *D. piscatorum* Prain & Burkill) is used as fish poison by the local people of Malaysia (Kumar et al., 2017). The tuber of *D. prazeri* is used to kill hair lice (Maneenoon et al., 2008). *D. hamiltonii* tubers are used as cooling agent during summer and used to cure diarrhoea (Dutta, 2015). The tuber juice of *D. wallichii* is used to treat stomach pain and jaundice (Rout and Panda, 2010). The tuber powder of *D. bulbifera* is applied in hernia and affected wound of scorpion bite (Nayak et al., 2004). In Jeypore tract of Odisha, the boiled tuber of *D. oppositifolia* is given to the mother after childbirth as body strengthener and *D. hamiltonii* is used to treat piles (Mishra et al., 2008). In Bangladesh, the tubers of *D. bulbifera* are used for the treatment of leprosy and tumor and in Chinese medicine these tubers are used against sore throat (Mbiantcha et al., 2011). In Zimbabwe, the tuber of *D. bulbifera* is used to cure wounds and sores, the bulbils paste are externally applied to boils and wound in Cameroon and Madagascar (Mbiantcha et al., 2011). The local tribal communities of Enugu in Nigeria used *D. alata* against fever and the tubers of *D. cayennensis* is used to treat diarrhoea (Aiyeloja and Bello, 2006). Different ethno-medicinal uses, their mode of preparation of *Dioscorea* are listed in **Table 1**.

**Table 1:** Ethno-medicinal information of different *Dioscorea* species.

Species	Ethno-medicinal uses	Mode of preparation/Doses	References
*D. bulbifera* L.	Piles, dysentery, and intestinal worm	Tuber paste is used orally for treatment of piles, dysentery, and worms.	Padhan and Panda (2016)
	Contraceptive	Tuber powder (10gm) is given once in a day for 5 days after menstruation.	Swarnkar and Katewa (2008)
	Kill hair lices	Tuber powder is applied with hair oil to kill the hair lices.	Padhan and Panda (2016)
	Cough and asthma	Boiled tuber is used to treat bronchial cough	Bhatt and Negi (2006)
	Typhoid	The tuber syrup with the turmeric powder is used to for a week to treat typhoid	Jain et al. (2008)
	Skin infection	Crushed tuber is applied to treat skin itching	Tiwari and Pande (2006)
	Body refrigerant heat during summer	Tuber and bulbils are eaten to reduce body heat during summer.	Sharma and Bastakoti (2009)
	Diarrhoea and dysentery	Tuber powder mixed with butter milk is given to check diarrhoea and dysentery	Jadhav et al. (2011)
*D. glabra* Roxb.	Piles	Tuber paste is used externally for treatment of piles	Padhan and Panda (2016)
	Health tonic	Tubers are eaten as health tonic for body strengthening	Mishra et al. (2008)
*D. hamiltonii* Hook.f.	Body refrigerant and in diarrhoea	Crushed tubers are applied as body refrigerant during summer seasons and good for treating diarrhoea.	Sharma and Bastakoti (2009)
	Stomach ache	Boiled tuber is consumed for treatment of stomach ache.	Edison et al. (2006)
	Piles	Boiled tuber is consumed to get relief from piles.	Mishra et al. (2008)
*D. hispida* Dennst.	Vomiting, indigestion	Boiled tuber is used to treat vomiting, indigestion	Dutta (2015)
	Wounds and injuries	Sap of tubers is applied around the affected parts and covered with clothes for one night to treat ‘wounds and injuries	Sharma and Bastakoti (2009)
	Eye treatment	Water of the soaked tuber is used to treat eyes	Meena and Yadav (2011)
	Fish poison	Crushed tuber are used as fish poison	Nashriyah et al. (2011)
*D. oppositifolia* L.	Joint swelling, scorpion and Snake bites	Tuber powder mixed with cow urine is applied on affected area of scorpion and snake bite.	Edison et al. (2006)
	Increase sperm number	Tuber powder mixed with honey is eaten.	Sharma and Bastakoti (2009)
	Antiseptic for ulcers	Leaf paste is used as antiseptic.	Sheikh et al. (2013)
*D. pentaphylla* L.	Abdominal pain after delivery	The tuber powder is given orally to treat abdominal pain after delivery. Decoction of tuber is given for curing asthma or cough.	Swarnkar and Katewa (2008)
	Joint swelling,	Tubers are applied on swelling of joints.	Edison et al. (2006)
	Body strengthener	Tubers are used as tonic to improve body immunity	Edison et al. (2006)
	Antiheminthic	Tubers are eaten to cure worm infection in stomach.	Sharma and Bastakoti (2009)
	Stomach troubles, rheumatic swellings	Tuber is used as tonic to cure stomach troubles and rheumatic swellings.	Dutta (2015)
*D. pubera* Blume.	Weakness	Tubers are eaten as tonic	Kumar and Satpathy (2011)
	Colic pain	Bulbils are cooked and eaten to cure colic pain.	Sheikh et al. (2013)
	Lactation of women	Lactating mothers are given 100–150g of boiled tuber with 250 g of black taro (*Colocasia esculenta*) to increase their milk flow.	Mishra et al. (2008)
*D. wallichii* Hook.f.	Jaundice	Cooked tubers are eaten and juice consumed for treatment.	Edison et al. (2006)
	Stomach pain	Boiled tubers are eaten.	Rout and Panda (2010)

## Pharmacological Studies of *Dioscorea* Species

*Dioscorea* species have been reported to have anti-microbial, anti-fungal, antimutagenic, hypoglycaemic, and immunomodulary effects (Kumar et al., 2017). The extracts of *Dioscorea bulbifera* and *Dioscorea alata* identified to have antifungal activities on *Botryodiploidia theobromae* (Eleazu et al., 2013). Several researchers have been validated the traditional knowledge by reporting the antimicrobial and anti fungal activities of wild yam *D. pentaphylla* against both gram positive and gram negative bacterias such as *Staphylococcus aureus*, *Pseudomonas aeruginosa, Streptococcus mutans*, *Streptococcus pyogenes, Vibrio cholera, Salmonella enteric-typhi, Shigella flexneri* and *Klebsiella pneumoniae*, and anti fungal activity against pathogenic fungi (Prakash and Hosetti, 2010; Kumar et al., 2013). Similarly, the leaf extract from *D. hamiltonii* also reported to have antimicrobial and antifungal potential against gram positive bacteria and fungi (Kaladhar et al., 2010). The silver nano-particles synthesized from *D.bulbifera* tuber extracts reported to possess potent synergistic antibacterial activity against both gram-negative and gram-positive bacteria (Ghosh et al., 2012). Researchers reported the analgesic and anti-inflammatory properties of the bulbils of *D. bulbifera* agaist paw oedema (Mbiantcha et al., 2011) and it has also anthelmintic activity against *Fasciola gigantica* and *Pheritima posthuma* (Adeniran and Sonibare, 2013).

The cytotoxicity effect of *D. alata* extract on human cancer cell lines has proven the presence of anticancerous components (Das et al., 2014). The wild yam species *D. oppositifolia* reported to have anti ulcer activity in adult wistar rats (Jhansi Rani et al., 2012; Mohan, 2012). Methanolic extract of *D. oppositifolia* reported to retarded the castor-oil induced intestinal transit and diarrhoea in rat (Jhansi Rani et al., 2012; Mohan, 2012). The anti-diabetic activities of *D. alata* (Maithili et al., 2011) and *D. bulbifera* (Ghosh et al., 2012; Okon and Ofeni, 2013) has been validated for management of type II diabetes.

## Food Value of *Dioscorea* Species

The inherent food quality trait of yam includes nutritional, anti- nutritional factors and physico-functional composition, which have significant utilization in human nutrition (Otegbayo et al., 2010). The health-promoting phytochemicals are referred as nutritional factors whereas the components have inhibitory effect on health are regarded as anti-nutritional factors. Understanding the necessity of these chemicals with their impacts on human health is the major challenges for consumers and researchers for implement them in yam improvement program. These phytochemicals should be highlighted in order to understand their beneficial or inhibitory effect on human health.

## Nutritional Parameters

Yams have been considered to have considerable amount of various dietary nutrients in comparison to other tropical tuber crops. The tuber of yams are reported to have good source of essential nutritional components such as starch, proteins, lipids, vitamins, and minerals, etc. (Arinathan et al., 2009; Mohan and Kalidass, 2010). The comparison of nutritional quality of yams with other tuber crops is presented in **Table 2**.

**Table 2:** Comparison of nutritional composition of selected crops with yam (USDA, 2015; Chandrasekara and Kumar, 2016).

Nutrient	Potatoes	Cassava	Sweet potatoes	Yams
**Proximate composition**				
Energy (kJ)	322	670	360	494
Protein (g)	2	1.4	1.6	1.5
Total lipid (g)	0.09	0.28	0.05	0.17
Carbohydrates (g)	17	38	20	28
Total dietary fiber (g)	2.2	1.8	3	4.1
Sugar (g)	0.78	1.7	4.18	0.5
**Minerals**				
Calcium (mg)	12	16	30	17
Iron (mg)	0.78	0.27	0.61	0.54
Magnesium (mg)	23	21	25	21
Phosphorus (mg)	57	27	47	55
Potassium (mg)	421	271	337	816
Sodium (mg)	6	14	55	9
**Vitamins**				
Vitamin C (mg)	19.7	20.6	2.4	17.1
Thiamin (B1) (mg)	0.08	0.09	0.08	0.11
Vitamin E (mg)	0.01	0.19	0.26	0.39
Beta-carotene (μg)	1	8	8509	83
**Fats**				
Saturated fatty acids (g)	0.03	0.07	0.02	0.04
Monounsaturated fatty acids (g)	0	0.08	0	0.01
Polyunsaturated fatty acids(g)	0.04	0.05	0.01	0.08

## Proximate Composition

Proximate composition is highly important in order to highlight the food quality and it includes moisture, ash, crude fat, crude protein, crude fiber, and carbohydrate (Polycarp et al., 2012). Moisture content of the food acts as an index to determine its water activity and food stability (Polycarp et al., 2012). Food with high moisture content is more susceptible to microbial contamination and food with low moisture content can be stored for long and suitable for processing in food industries (Otegbayo et al., 2018). Dehydration in food leads to increase in other food nutrients and also improve the shelf life during food preservation (Otegbayo et al., 2018). The moisture content varied from 58 to 79% in Ghanaian yams (*D. rotundata, D. bulbifera, D.cayennensis, D. dumetorum, D. alata*, and *D. esculenta*) (Polycarp et al., 2012), 71 to 92% in Indian yams (*D.alata, D. bulbifera, D. esculenta, D. oppositifolia, D. pentaphylla, D. tomentosa*, and *D. wallichii*) (Shanthakumari et al., 2008) and 19 to 30% in Nepalese wild yams (*D. bulbifera, D. versicolor, D. deltoidea*, and *D. triphylla*) (Bhandari et al., 2003).

The ash content of the food determines the presence of important dietary minerals and useful for the development of the body (Otegbayo et al., 2018). The ash content of yam is lower than the other tuber crops like potato and cassava (Bhandari et al., 2003; Otegbayo et al., 2018). Dietary fats help in absorption and retention of flavors during cooking which leads to increased palatability of food (Otegbayo et al., 2018). The dietary fats contributed 1%–2% of the food calorific value which is sufficient for the diet (Otegbayo et al., 2018) and the dietary fat or lipid content of yam has been reported to be higher than potato and sweet potato (Otegbayo et al., 2018). The dietary fiber of food protects the beneficial microflora of the intestine, also reduces the threat of colon cancer and cardiovascular diseases (Otegbayo et al., 2018). The high fiber in the diet improves the digestion and absorption process of large intestine, which helps to prevent constipation (Baah et al., 2009). Researchers have reported that yam species contains more amount of dietary fiber than other tuber crops such as potatoes, cassava and sweet potatoes (Shanthakumari et al., 2008; Baah et al., 2009).

Protein helps in the structural and functional activities of cell as well as to regulate the metabolic activities in all living organisms. Proteinaceous diet is essential in the daily diets of human beings (Natesh et al., 2017). Sufficient protein amount in the diet leads to increase the calorific value of the food and it is a reflection of a nutritionally satisfactory diet (Polycarp et al., 2012). If the protein content contributed 12% of the total calorific value of the food then it considered as good source of protein diet (Otegbayo et al., 2018). The yam species are reported to have higher protein content than other important tuber crops like cassava (Charles et al., 2005) and sweet potato (Moongngarm, 2013). Researchers from different parts of the world reported varying proportion of protein in yam species *viz*. Ethiopian yam (*D. bulbifera*) (9.7%) (Tamiru et al., 2008); Sri Lankan yams (*D. alata* and *D. esculenta*) (10.16%) (Senanayake et al., 2012); some Indian varieties (*D.alata, D. bulbifera, D. esculenta, D. oppositifolia, D. pentaphylla, D. tomentosa*, and *D. wallichii*) (15.75%) (Shanthakumari et al., 2008); Ghanaian yams (*D. rotundata, D. bulbifera, D.cayennensis, D. dumetorum, D. alata*, and *D. esculenta*) (Polycarp et al., 2012), and 5.3% for Indonesian yams (*D.alata* var Krimbang) (Aprianita et al., 2014).

Carbohydrate is an integral part of proximate composition of food that provides energy to the body and plays a pivotal role in structure and function of cellular mechanism (Baah et al., 2009). It increases the nutritional value of the food as well as improves the organoleptic properties of the food (Polycarp et al., 2012). Sugar and starch improve the palatability and texture of the food, which influence the food preference. Starch content of different plants are varying due to the differences in enzymatic activities for its biosynthesis process (Otegbayo et al., 2018). The sugar and starch content of the yams has been reported to be less than the potatoes and cassava (Baah et al., 2009; Afoakwa et al., 2013; Otegbayo et al., 2018). The high content of non-starchy carbohydrates in the food is due to the presence of high dietary fiber, which has an important role as a functional food (Otegbayo et al., 2018). The other non-starchy carbohydrates such as lignin, cellulose, and hemicelluloses also significantly influence the texture quality of yam (Otegbayo et al., 2018). Yams has been reported to provide 12% of the energetic food for the people of tropical countries after cassava (20%) and followed by taro (4%) and sweet potato (2%) (Otegbayo et al., 2018).

## Vitamins

Different dietary vitamins help to use protein, fat and carbohydrate to make energy and available to the body. The vitamins C and E are considered as antioxidant and act as cofactors for enzymes. Vitamin C has multiple functions as radical scavenging activities, collagen synthesis, iron absorption, wound healing properties, and anti-inflammatory activities. The yam tubers contain different vitamins higher than other tuber crops (USDA, 2015). Vitamin C is the most abundant vitamins in yam tubers (Udensi et al., 2008). The most widely cultivated species viz. *D. cayennensis* is reported to have higher carotenoid content (Bhattacharjee et al., 2011).

## Minerals

Dietary minerals are essential for the diet of human beings that plays a vital role in various metabolic process of the body (Polycarp et al., 2012). Calcium is an essential mineral, which helps in coagulating blood and maintain the integrity of intracellular cementing materials (Polycarp et al., 2012). Iron is an integral part of formation of blood hemoglobin and helps in transportation of oxygen in the body. The deficiency in iron content in the body causes myocardial diseases, gastrointestinal infection, nose bleeding, etc. (Polycarp et al., 2012). Zinc is an essential mineral that plays a pivotal role in development of brain and bone and also wound healing capacity (Padhan et al., 2018). It also helps in metabolic activities of carbohydrate, protein, vitamin A, and nucleic acid biosynthesis process (Padhan et al., 2018). High potassium content in the body increases the iron utilization which is beneficial for controlling hypertension (Padhan et al., 2020). The amount of potassium is beneficial for the diuretics people to control hypertension (Padhan et al., 2020). The yam tubers are rich in dietary minerals and among all the minerals potassium is the abundant mineral present in the yam tubers (Baah et al., 2009; Polycarp et al., 2012).

## Physico-Functional Properties

Physico-functional properties are the basic biochemical properties that reveal the relationship between the structural and functional properties of food (Afoakwa et al., 2012). The functional parameters provide information on how food ingredients behave in a food system during processing (Afoakwa et al., 2012). The physico-functional properties such as water absorption capacity (WAC), foam capacity (FC), paste clarity (PC), water solubility index (WSI), and iodine affinity to starch (IAS), bulk density, and gelatinization temperature are the important parameters in food industries for bioprospecting of food ingredients (Afoakwa et al., 2012). Different factors influence the physico-functional properties which include starch and ratio of amylose to amylopectin (Sanful et al., 2013). In *Dioscorea* tuber various physico-functional parameters have been studied by many researchers and stated the use of the yam flour for making food products (Sanful et al., 2013; Ojinnaka et al., 2017).

The bulk density of the flour reflects the relative volume of packaging material. Lower bulk densities are more desirable as this imply the sample would pack better during storage or distribution. Flour with high bulk density is better for formulation of complementary foods (Chandra et al., 2015). Water absorption capacity (WAC) is the amount of water absorbed by flour to produce dough of required consistency (Chandra et al., 2015). Interactions of the flour with water and oil reflect their effects on the flavor and texture of foods. High water absorption may assure the product cohesiveness and this is a functional characteristic mostly important for ready-to-use foods but may also be important for dough making. The higher WAC of the flour is due to the presence of hydrophilic constituents such as polysaccharides, polar amino acids, and increase in the amylose leaching and loss in integrity of starch structure. The flour with high WAC is suitable in formulation of some food products and bakery products where viscosity is required (Chandra et al., 2015). The water solubility index (WSI) is related to amylose leaching from starch granules (Moorthy, 2002).

Foam in the food improves the texture, consistency and appearance of food product (Chandra et al., 2015). The foam capacity (FC) indicates the amount of interfacial area created by the protein present in the flour (Chandra et al., 2015). The foam capacity is inversely related to the foam stability (Chandra et al., 2015). Flours with high foaming capacity could form large air bubbles surrounded by thinner and less flexible protein film. Paste clarity (PC) is a desirable property that influences the brightness and turbidity of the food (Mweta et al., 2008). The increase in transmittance of paste is due to the gelatinization of starch of the flour and the paste obtained after gelatinization are more transparent than the suspension (Nuessli et al., 2000). As per the study of Padhan et al. (2020) the wild species such as *D. hamiltonii*, *D. pubera* and *D. oppositifolia* had higher values of WAC, FC, PC, WSI, and IAS than that of cultivated species *D. alata.* They suggested these wild yam tuber flours, have a good potential to be used as a food ingredient in the food industry. The gelatinization temperature is one of the physico-functional properties of flour that refers the temperature required to gelatinize the starch (Nuessli et al., 2000). The flour with higher starch content took lower temperature for gelatinization.

## Anti-Nutritional Factors in *Dioscorea* Species

Anti-nutritional factors are the naturally occurring chemical compounds synthesized by normal metabolism which reduces the nutrient utilization by the body (Bhandari and Kawabata, 2004). Anti-nutritional factors affect the bioavailability of dietary nutrients especially protein, minerals, and vitamins and reduce the nutritive value of the food (Padhan et al., 2018). The tubers of yam species are acrid which contain different anti-nutritional factors associated with skin irritation and inflammation of the buccal cavity and throat after consumption (Kumar et al., 2017). The phenol, alkaloid, oxalate, phytate, tannin, saponin, amylase inhibitor, trypsin inhibitors are considered to be the anti-nutritional factors in yams which are responsible for toxicity and bitterness (Poornima and Ravishankar Rai, 2009).

Alpha amylase inhibitors alter the catalytic action of alpha amylase enzyme on starch and consequently slow down or stop the breakdown of starch to maltose (Agarwal and Jain, 2010). These are the glycoproteins with molecular weights in the range of 45,000–49,000 kDa (Jamil et al., 2000). The inactivation of amylase enzyme reduces the starch digestion (Jamil et al., 2000). The amylase inhibitor forms complex of equal ratio (1:1) with pancreatic amylase enzyme and binds at the site other than the active site of the enzyme thus inactivating the catalytic power of the enzyme through conformational changes (Jamil et al., 2000). The presence of alpha amylase content in yam tubers is more than the other commercial tuber crops (Kaur et al., 2011; Polycarp et al., 2012; Padhan et al., 2020).

Trypsin inhibitor belongs to a broad class of proteins (protease inhibitors) that inhibit proteolytic enzymes. Trypsin inhibitor is a protease inhibitor that inhibits the enzymatic activities of trypsin and chymotrypsin in the digestive tract, thus forming indigestible complexes with dietary protein and impairing protein digestion (Bhandari and Kawabata, 2006). The trypsin inhibitors content has been reported to be more in wild yam tubers than the cultivated species (Bhandari and Kawabata, 2006; Padhan et al., 2020).

Alkaloids are the largest group of secondary metabolites that comprises of nitrogen bases synthesized from amino acids. The derivatives of alkaloids have various pharmacological importance such as analgesic, antispasmodic, and antibacterial properties (Polycarp et al., 2012). Wild yam species are reported to contain more alkaloid content than the cultivated species (Polycarp et al., 2012). Flavonoids are reported to be the most abundant polyphenols in human diets (Yunfeng et al., 2006). Flavonoids are structurally made of more than one benzene ring (Yunfeng et al., 2006). Flavonoids in combination with the positively charged amino acids and dietary minerals such as iron, zinc, and calcium reduces the bioavailability of dietary minerals (Bhandari and Kawabata, 2004). These are potent water-soluble antioxidants and responsible for anti-microbial, anti-inflammatory properties, and anti-carcinogenic activities (Bhandari and Kawabata, 2004). The flavonoid content in yam species are reported to have the antioxidant capacity to scavenge the free radicals (Bhandari and Kawabata, 2004).

Tannins are responsible for the astringent taste of foods and drinks. It forms complexes with dietary protein of the food and results in precipitation of protein and impair its availability (Natesh et al., 2017). Higher concentration of tannin affects the protein quality of the food and interferes with iron absorption (Padhan et al., 2020). The plants rich in tannin content have been reported for the treatment of diseases like leucorrhoea, rhinnorhoea, healing of wounds, and diarrhoea (Eleazu et al., 2013). The bitterness of the yam species is due to the presence of tannins in them (Padhan et al., 2020).

Saponins are naturally occurring compounds which are made up of sugar molecule in combination with triterpene or steroid glycone (Eleazu et al., 2013). The steroidal and triterpene are two major types of saponin. A higher concentration of saponins causes hemolysis of blood, but they also have pharmacological potentials such as cholesterol lowering and anti-cancerous activities. Steroidal saponins have been reported to be the major physiologically active constituents in yams (Avula et al., 2014). A total of 21 steroidal saponins with six aglycone skeletons were identified from the methanolic extracts of *D. villosa* and *D. cayenensis* (Avula et al., 2014).The wild yam species *D. bulbifera* contain saponins which has hemolytic activity, antimicrobial activities, and cholesterol binding properties (Okigbo et al., 2009). *Dioscorea* species have 20 different types of steroidal saponins that have various pharmacological properties (Avula et al., 2012). The saponins from yam species have been used in industries for making steroid drugs (Kumar et al., 2017). Over 50 steroid saponins of furostan-, spirostan-, and pregnane-type skeletons have been reported to be the major physiologically active constituents from various *Dioscorea* species (Benghuzzi et al., 2003). Sautour et al. (2006) reported six saponins from wild species of yam native to North America, *D. villosa* such as protodioscin, methyl protodioscin, parrisaponin, dioscin, pro-genin III (prosapogenin A of dioscin), and proge-nin II. Hayes et al. (2007) identified four major and three minor steroidal saponins from *D. villosa* using 2D NMR spectroscopy. The major saponins are two furanostane types, methyl parvifloside, and protodeltonin, as well as two spirostane types, deltonin, and glucosidodeltonin (zingiberensis I) and the minor saponins included methylprotodioscin, disoscin, and prosapogenin A of diosgenin. Teponno et al. (2006) isolated pennagenin Spiroconazole A, a steroidal saponin from the tuber of *D. bulbifera*.

Phenolic compounds have an inhibitory effect on plant growth. They are usually combined with glucosyl residues within plant tissue. Phenols are designated as anti-nutrients because they decrease the digestibility of proteins, carbohydrates, and minerals and thus, make them insoluble (Padhan et al., 2018). They also inhibit the activity of digestive enzymes such as amylase, trypsin, and chymotrypsin thereby, causing damage to mucosa of digestive tract (Bhandari and Kawabata, 2004). The phenols from the yam species are the major cause of browning of the tuber flesh when it is exposed to the air (Bhandari and Kawabata, 2004). Researchers stated that the presence of phenol content in yam species contributed to the antioxidant capacity (Bhandari and Kawabata, 2004; Niu et al., 2010; Cornago et al., 2011). Teponno et al. (2006) identified four polyphenolic compounds from the tuber of *D.bulbifera* such as dihydroxy-4- methoxy phenanthrene, quercetin, quercetin-3-O-β-D-glucopyranoside, and quercetin-3-O- β– D-galactopyranoside.

Phytate is the salt form of phytic acid, primarily present as the mono or divalent cations with K^+^, Mg^2+^, and Ca^2+^ (Padhan et al., 2018). Phytate is the storage form of both phosphate and inositol in plant seeds, tubers, and grains. Phytate has a negative impact on the bioavailability of dietary minerals such as zinc, iron, calcium, copper, and magnesium that led to the mineral deficiency in the body (Padhan et al., 2018). The presence of phytate in yam species was reported to be more than the other tuber crops (Polycarp et al., 2012; Padhan et al., 2018).

Oxalate is present in the form of calcium oxalate and widely distributed in plants. The oxalic acid strongly bonds with the dietary minerals such as Ca, Mg, Na, and K and resulted in the formation of oxalate salts (Padhan et al., 2018). The insoluble calcium oxalate salts precipitate in kidney and urinary tract and forms calcium oxalate crystals that cause kidney stones (Padhan et al., 2018). Higher oxalate concentration in food causes nutritional deficiency and severe throat irritation. The yam mucilage causes skin and mucous membrane irritation due to the presence of calcium oxalate crystals (raphides) (Otegbayo et al., 2018). Wild yam species have been reported to contain a greater number of oxalates that cause skin irritation and inflammation of throat (Bhandari and Kawabata, 2006; Polycarp et al., 2012).

## Bioactive Components IN *Dioscorea* Species

The bioactive components are the plant based secondary metabolites used in defense mechanism against various insects and pests. These bioactive components such as phenols, polyphenols, alkaloids, polypeptides, steroids, terpenoids, and essential oils have various pharmacological activities (Alamu et al., 2014). The *Dioscorea* species are known to contain a good quantity of bioactive compounds such as phenols, alkaloids, tannins, flavonoids, saponins, glycoside steroids, anthraquinones, etc. (Price et al., 2017). The tubers of different *Dioscorea* species reported to have various bioactive compounds and the details are presented in **Table 3**.

**Table 3:** Different bioactive compounds present in *Dioscorea* species.

Species	Bioactive components	Supporting literatures
*D. alata* L.	Dioscorins, diosgenin, water soluble polysachharides	Harijono et al. (2013)
*D. alata* L.*, D. bulbifera* L.*, D. cayennensis* D. cayennensis Lam. subsp. cayennensis*, D. dumetorum* (Kunth) Pax	Beta-carotene, Mutatochrome, Lutein Neoxanthin, Violaxanthin, Zeta-carotene, Phytoene, Antheraxanthin, Beta-cryptoxanthin, Zeaxanthin, C25-epoxy-apocarotenoid persicaxanthin	Price et al. (2018)
*D. bulbifera* bulbil, *D. cayennensis* Lam.	Allantoin	Lebot et al., 2019
*D. dumetorum* (Kunth) Pax	β-carotene epoxides, mutatochrome	Ferede et al. (2010)
*D. opposita* Thunb.	Soluble proteins and mannanin mucilage	Myoda et al. (2006)
*D. pseudojaponica* Thunb.*, D. batatas* Decaisne	Allantoin and allantoic acid	Yi et al. (2005)
*D. villosa* L.	Protodioscin, methyl protodioscin, parrisaponin, dioscin, pro-genin III (prosapogenin A of dioscin), pro-genin II	Sautour et al. (2006)
*D. villosa* L.	Furanostane, methyl parvifloside, protodeltonin, deltonin and glucosidodeltonin (zingiberensis I)	Hayes et al. (2007)
*D. villosa* L.	Cholestane steroid glycosides	Ali et al. (2013)
*D. villosa* L.	dioscoreavilloside A and B, parvifloside	Avula et al. (2014)
*D. zingiberensis* C.H.Wright	Zingiberensis saponin I, deltonin, gracillin, dioscin, asperin, and pro-genin III	Zhu et al. (2010)
*D. zingiberensis* C.H.Wright, *D. septemloba* Thunb., *D. collettii* var. hypoglauca (Palib.) S.J.Pei & C.T.Ting	Diosgenin	Yi et al. (2014)

Diosgenin is a steroidal sapogenin (C_27_) that belongs to the triterpene group and the typical bioactive compound of *Dioscorea* family (Shah and Lele, 2012). Around 15 species of *Dioscorea* are used as a source of diosgenin, with an estimated market value of $500 million (Price et al., 2017). Three sapogenins have been isolated from yam species are diosgenin, botogenin, and kryptogenin (Shah and Lele, 2012). Diosgenin from *Dioscorea* species serves as a precursor for the production of steroid drugs such as cortisone (Shah and Lele, 2012). It is reported to decrease cholesterol absorption and prevent colon cancer (Shah and Lele, 2012). The diosgenin have various pharmacological activities such as antimicrobial and anti-inflammatory activities (Zhang et al., 2014). China and Mexico are the richest diosgenin producer in the world and contributed 67% of world diosgenin production (Li et al., 2010; Yi et al., 2014). Yi et al. (2014) reported diosgenin from three *Dioscorea* species namely *D. zingiberensis* C.H.Wright, *D. septemloba* Thunb., *D. collettii* var. hypoglauca (Palib.) S.J.Pei & C.T.Ting. Asha and Nair (2005) also reported that some wild *Dioscorea* species such as *D. pubera* and *D. spicata* contains maximum diosgenin yield. In India, about 800 to 900 tonnes of dry rhizomes of *Dioscorea deltoidea* has been exploited annually, as the demand for diosgenin is increasing in the Indian pharmaceutical industry (Shah and Lele, 2012).

*Dioscorea* species reported to contain other bioactive compounds such as water-soluble polysaccharides and dioscorin (Myoda et al., 2006; Shah and Lele, 2012; Harijono et al., 2013). Dioscorin is a storage protein of yam species which act as trypsin inhibitor, carbonic anhydrase, antioxidant, immunomodulator, and hypertension invasion (Hou et al., 2001; Liu et al., 2007). Dioscorin accounts for over 90% of the extractable proteins in yam (Rachman, 2011). So far, the dioscorin content has been reported from some of the *Dioscorea* species only such as *D. opposita, D. alata, D. japonica, D. esculenta*, and *D. batata* (Liu et al., 2007; Harijono et al., 2013).

Some of the active constituents of yams are gradually gaining attention not only for their nutritive value but also for their medicinal properties (Lee et al., 2010). Allantoin and dioscin are also well known active constituents from tubers of *Dioscorea* species (Wei et al., 2013). The considerable amount of allantoin and dioscin has been reported in different cultivated germplasm of *Dioscorea* species in China (Fu et al., 2006; Wu et al., 2016). The allantoin of the yam species is responsible for α-amylase and α-glucosidase activity that act as antidiabetic properties as well as antioxidant and anti-dyslipidemic activities (Niu et al., 2010).

Water- soluble polysaccharides (WSP) are another bioactive component from yellow and white water yam reported to reduce the blood glucose and cholesterol levels especially the LDL cholesterol due to the presence of glucomannan (Harijono et al., 2013). The WSP of *D. opposita* reported to have hyperglycemic properties (Fan et al., 2015). The WSP also has the ability to improve the body immune system. Fan et al. (2015) reported the increase in lymphocyte, macrophage and natural killer cell after administration of WSP extract of *D. opposita*. Many researchers highlighted the pharmacognostical and phytochemical studies of various species of *Dioscorea* such as *D. oppositifolia*, *D. bulbifera*, and *D. alata* and reported the presence of alkaloids, amino acids, flavonoids, steroids and triterpenoids, tannins, and saponin (Jhansi Rani and Mohana, 2012; Azeem et al., 2013; Eleazu et al., 2013).

## Antioxidant Status of *Dioscorea* Species

Free radicals are generated during various metabolic processes that have deleterious effects on degradation of nucleic acid, proteins and lipid peroxidation activities etc. (Ekrem and Llhami, 2008). Various reactive oxygen species (ROS) such as superoxide anion, hydroxyl radical, and hydrogen peroxide degrade cell membranes and destroy cells which eventually causes different degenerative diseases (Ekrem and Llhami, 2008). Studies in yams indicated low to high content of polyphenol and antioxidant activities (Kaur and Kapoor, 2002; Fang et al., 2011). The antioxidant activity of phenolic compounds is mainly due to their redox mechanisms like singlet oxygen quenching ability, radical scavenging activity, and metal chelating activity. (Ekrem and Llhami, 2008). Yams also contain other antioxidants such as vitamin C and carotenoids which exerts useful physiological effects (Champagne et al., 2010; Ferede et al., 2010). Narkhede et al. (2013) investigated the antioxidant capacity of *D. alata* by taking water and ethanol extract and reported that both the extracts showed high free radical scavenging potential. Similarly, *D. alata* also showed effective reductive potential against 2, 2-diphenyl-1-picrylhydrazyl (DPPH), nitric oxide, and lipid peroxidation. Sakthidevi and Mohan (2013) reported that the methanol extract of *D. alata* had potential to scavenge hydroxyl, superoxide, ABTS^+^ radicals whereas ethanol extract of tuber showed strong DPPH radical scavenging activity. Lubag et al. (2008) investigated the antioxidant analysis of nine cultivars of greater yam (*D. alata*) from the Philippines and reported that cultivars of greater yam (*D. alata*), with color ranging from white to intense purple, had high antioxidant activities similar or higher than the control BHA (butylhydroxy anisole) and α-tocopherol. Different types of antioxidant activity assays has been used by many researchers to determine the antioxidant activities of yam species (Lin et al., 2005; Cornago et al., 2011; Ghosh et al., 2013; Narkhede et al., 2013; Ukom et al., 2014). Bhandari and Kawabata (2004) also reported considerable antioxidant activity in wild yam tubers from Nepal using DPPH assay and revealed the relationship with the total polyphenols and flavonoids to the antioxidant activity of the yam (Cornago et al., 2011; Ukom et al., 2014). According to the study of Ghosh et al. (2013), the bulbil of *D. bulbifera* showed high scavenging activities against pulse radiolysis generated OH^·^ radicals and ABTS+ radicals and they stated that the species could be used as a potential source for herbal therapeutic agents against various diseases caused by oxidative stress.

## Conclusion and Future Remarks

Yams regarded as an energy contributor provide a number of desirable nutritional components and health benefits such as antioxidative, hypoglycemic, hypocholesterolemic, antimicrobial, and immunomodulatory activities. These wild yam tubers may serve as functional food and nutraceuticals for treatment of chronic diseases. Research should be carried out to utilize the bioactive compounds present in these tubers for formulation of new drugs to fight against different diseases. The physicofunctional capacity of wild yams need to be explored for utilisation of yam flours in food systems and other industrial applications. Attempt should also be made toward *in vitro* trails of the bioactive components derived from wild yams using animal models in order to pin down their potential applications in drug discovery for curing various life threatening diseases. The yam genetics and genomics need to be addressed in the near future for crop improvement of these wild species. Candidate gene identification using microarray and other molecular approaches has to be conducted in order to identify QTLs involved in important nutritional traits. Future research is needed to better understanding of the phenotypic characteristics of wild species to explore the hidden genetic potential for biodiversity management for sustainable development and germplasm conservation.

## Author Contributions

BP and DP designed and wrote the paper. All the authors read and provided helpful discussions for the manuscript.

## Conflict of Interest

The authors declare that the research was conducted in the absence of any commercial or financial relationships that could be construed as a potential conflict of interest.
